# Maximising antihypertensive effects of angiotensin II receptor blockers with thiazide diuretic combination therapy: focus on irbesartan/hydrochlorothiazide

**DOI:** 10.1111/j.1742-1241.2007.01577.x

**Published:** 2007-12

**Authors:** J M Flack

**Affiliations:** Department of Internal Medicine, Wayne State University and the Detroit Medical Center Detroit, MI, USA

## Abstract

**Background:**

Evidence-based guidelines for the management of hypertension are now well established. Studies have shown that more than 60% of patients with hypertension will require two or more drugs to achieve current treatment targets.

**Discussion:**

Combination therapy is recommended as first-line treatment by the JNC-7 guidelines for patients with a blood pressure > 20 mmHg above the systolic goal or 10 mmHg above the diastolic goal, while the International Society of Hypertension in Blacks recommends combination therapy when BP exceeds targets by > 15/10 mmHg. Current European Society of Hypertension-European Society of Cardiology guidelines also recommend the use of low-dose combination therapy in the first-line setting. Furthermore, JNC-7 recommends that a thiazide-type diuretic should be part of initial first-line combination therapy. Thiazide/diuretic combinations are available for a variety of classes of antihypertensive, including ACE inhibitors, angiotensin receptor blockers (ARBs), beta blockers and centrally acting agents. This article focuses on clinical data investigating the combination of an ARB, irbesartan, with the diuretic, hydrochlorothiazide.

**Conclusions:**

These data indicate that the ARB/HCTZ combination has greater potency and a similar side effect profile to ARB monotherapy and represents a highly effective approach for attaining goal BP levels using a therapeutic strategy that very effectively lowers BP, is well tolerated and minimises diuretic-induced metabolic effects.

**Review Criteria**

The PubMed and other searchable databases were utilized to collate information from original and review articles as well as from selected abstracts relevant to this topic.

**Message for the Clinic**

Diuretic-based combination antihypertensive drug therapy is a cornerstone of antihypertensive drug therapy. Most hypertensive patients will require more than one antihypertensive drug to lower blood pressure (BP) below target levels. The combination of diuretics with renin angiotensin system antagonists is highly logical given the significant augmentation of BP response and the minimization of drug-specific side effects (e.g., hypo- and hyperkalemia) when these two drug classes are combined. The combined use of angiotensin receptor blockers and diuretics is better tolerated, but more costly, than generic angiotensin converting enzyme inhibitors and diuretics, mostly because of the absence of cough and much lower incidence of angioedema.

## Introduction

Hypertension affects almost 29% of the adult US population, an estimated 58.4 million individuals (1). Worldwide, hypertension may affect as many as 1 billion individuals, with approximately 7.1 million deaths per year attributable to the condition (2). The prevalence of hypertension increases with advancing age to the point where more than half of the people aged 60–69 years of age and approximately three-quarters of those aged 70 years or older are affected (2). As a major risk factor for cardiovascular disease, stroke, retinopathy, and renal failure, hypertension has major global public health implications, and the challenge of achieving effective blood pressure (BP) control is growing in importance as populations age throughout the world.

Maintaining aggressive BP targets is the basis of preventing the long-term adverse outcomes of hypertension. The linkage of efficacious and prompt treatment has been suggested by the results of the VALUE trial, which was designed to compare the incidence of cardiac morbidity and mortality when the same level of BP control was achieved using treatment regimens based on the angiotensin receptor blocker (ARB) valsartan or the calcium channel blocker amlodipine (3). However, BP was in fact controlled more rapidly and to a slightly greater degree in the amlodipine arm during the early months of the trial, and this difference was correlated with a significantly higher incidence of myocardial infarction and a trend towards a higher incidence of stroke in the valsartan group where BP was less effectively controlled (3). The time relationship of excess events in the valsartan group compared with amlodipine can best be explained by the between-group differences in BP, which were largest in the first year. Overall, 63% of the entire observed excess of strokes occurred in the first 6 months, and 76% by the end of the first year (3,4). These data might be interpreted as showing that the speed of attaining BP targets is important; however, it is also likely that the withdrawal of antihypertensive drug therapy from stable but severely hypertensive patients, followed by subsequent randomisation to monotherapies that differentially controlled BP early on, contributed to the higher event rate early in the VALUE trial in the valsartan compared with the amlodipine treatment arms. Nevertheless, therapeutic inertia contributes to slow control of BP to target levels and plausibly contributes to a modest augmentation of risk for pressure-related events even over the short-term.

While BP goal attainment is the most crucial objective of antihypertensive treatment, other considerations are also of fundamental importance. One key consideration is that BP control should be sustained over 24 h. Long-acting antihypertensive agents provide 24-h BP control from a single daily dose, as well as attenuating the early morning rise in BP (5). Long-acting, once-daily antihypertensive drugs also provide greater protection against a rise in BP after missed medication doses in intermittently non-compliant patients. Treatment regimens should be designed to support long-term treatment adherence. In this regard, issues of patient education and the physician-patient relationship are critical (6). When regarding drug selection it is important to prescribe the simplest possible dosage (7). Several studies have found that once-daily dosage regimens are associated with better compliance than twice-daily regimens (8). Within the context of combination therapy, fixed-dose combinations in which treatment is administered as a single daily pill enable the simplicity of treatment to be sustained with a lower overall pill burden to the patient. Tolerability is also crucial to adherence (7). Although hypertension is generally considered to be asymptomatic, strong evidence suggests this assumption is incorrect (9,10). The use of antihypertensive medications that compromise quality of life can be especially troubling for many patients with uncomplicated hypertension. This may result in discontinuation of therapy (11). Indeed, in one study the number of adverse events associated with antihypertensive therapy, and not the achieved BP reduction, was found to directly correlate with changes in quality of life (12). In summary, treatment should be effective, with rapid and aggressive attainment of goal BP levels that are maintained throughout the entire day and night. Additionally, antihypertensive treatment regimens should be simple, convenient and well tolerated, and patients should be supported appropriately to sustain treatment adherence long term. Combination therapy clearly helps to attain all of these desirable aims.

## Meeting BP targets with combination therapy

More than two-thirds of hypertensive individuals will require two or more antihypertensive agents selected from different drug classes to achieve their BP targets (13,14). For example, in the ALLHAT study, 60% of those whose BP was controlled to < 140/90 mmHg received two or more agents, whereas only 30% overall were controlled on one drug (14). In the Hypertension Optimal Treatment (HOT) study, only 37% of patients reached the target diastolic BP < 90 mmHg with monotherapy (15). Clinical data show that patients with diabetes mellitus or renal disease will require greater intensity of antihypertensive treatment to attain their, albeit lower, BP goals. Accordingly, these hypertensives will require an average of 2.6 to 4.3 different antihypertensive medications to achieve a BP goal of lower than 130/80 mmHg (16). Both the JNC-7 and the 2003 European Society of Hypertension-European Society of Cardiology guidelines acknowledge that combination therapy may be necessary and, indeed, first-line combination treatment is recommended in JNC-7 for patients with a BP > 20 mmHg above the systolic goal or 10 mmHg above the diastolic goal (2,17). Hypertension guidelines from the International Society on Hypertension in Blacks (ISHIB) suggest the use of combination therapy when BP is > 15 mmHg above the systolic goal and/or > 10 mmHg above the diastolic goal (16).

JNC-7 recommends that combination therapy should usually include a thiazide-type diuretic as first-line therapy for stage 2 hypertension as well as for patients with compelling indications (2). Accordingly, this article focuses on combination therapy based on hydrochlorothiazide (HCTZ), specifically in combination with ARBs. It should be borne in mind, however, that combination therapy without thiazides is also an option as the combination of a calcium channel blocker with an ACE inhibitor or an ARB lowers BP very effectively (18,19). Equally, thiazides may be combined with a range of agents in addition to the ARBs, including beta blockers, centrally acting agents and ACE inhibitors (20–22). Each of these combinations may be appropriate in selected patients and treatment should be devised on a personalised, case-by-case basis.

## ARBs: clinical potential and combination with hydrochlorothiazide

Angiotensin receptor blockers have an established record of BP-lowering efficacy and a placebo-like side effect profile in hypertensive patients. Recent outcome studies in high-risk hypertensives have shown that ARBs provide cardiovascular-renal protection beyond what can be entirely attributed to BP-lowering alone. In hypertensives with ECG-LVH, there was a lower incidence of stroke in the losartan (ARB) than the atenolol (beta-blocker) treatment arm despite attainment of virtually identical BP lowering as determined by cuff measurements (23). Losartan-based therapy also proved superior to a treatment regimen that did not include either ACE inhibitors or ARBs in slowing the progressive loss of kidney function in patients with diabetic nephropathy (24). Irbesartan was shown to protect against progressive loss of kidney function and congestive heart failure as well as to reduce proteinuria more than amlodipine, or a placebo-based (no ACE, ARB or CCB) treatment regimen in hypertensives with diabetic nephropathy (25,26). Valsartan has been shown to improve outcomes in heart failure and postmyocardial infarction (27,28), while candesartan reduces the incidence of stroke and protects against mortality and cardiovascular events in patients with heart failure (29–32). Angiotensin receptor blockers also lower the incidence of new-onset diabetes (33) and atrial fibrillation (34).

Notwithstanding these benefits, ARBs like other classes of antihypertensive do not provide sufficient BP control for many patients when used in monotherapy. Indeed, ARBs, similar to ACE inhibitors, have near-flat dose–response curves suggesting that monotherapy dose titration offers limited benefits. This was shown in a pooled meta analysis of 43 published randomised clinical trials of losartan, valsartan, irbesartan and candesartan when administered at doses recommended for the treatment of hypertension (35). ARB monotherapy dose escalation resulted in only a modest incremental diastolic BP reduction compared with the starting dose of ARB. However, the near-flat dose response was resolved by the combination of ARBs with low-dose diuretics which significantly potentiated the BP reduction.

In addition to their impressive combined BP-lowering effects, the use of ARBs and HCTZ in combination counteracts the potential adverse effects of these agents when given as monotherapy. Because of the tendency of ARBs to elevate potassium levels, this is less likely to be a problem when combined with diuretics (36). Likewise, many of the undesirable metabolic side effects of thiazide monotherapy, including hypokalaemia, and elevated serum levels of uric acids, lipids and blood glucose levels, are minimised by the addition of an ARB (21,23,37,38).

Tolerability considerations also support the use of ARBs rather than ACE inhibitors in combination therapy. For instance, in the recent Blood Pressure Reduction and Tolerability of Valsartan in Comparison With Lisinopril (PREVAIL) trial, patients with mild-to-moderate hypertension treated with ARB/HTCZ combination were less likely to experience adverse events than those treated with ACE-inhibitor/HTCZ combination therapy (5.1% vs. 10.7%, p = 0.001). Drug-related dry cough also occurred more frequently in the patients receiving ACE inhibitor (7.2%) than in those receiving ARB (1.0%) (39). Angioedema, a serious adverse event which can be life-threatening (40), is experienced much less frequently as a side effect of ARBs, as demonstrated in the LIFE and VALUE studies (22,23). Hyperkalaemia has been less common with ARBs than ACE inhibitors in patients with reduced kidney function (41). These tolerability advantages must be weighed against the likely lower cost of generic ACE inhibitors, which provide a viable alternative HCTZ combination for patients able to tolerate long-term treatment.

**Figure 1:**
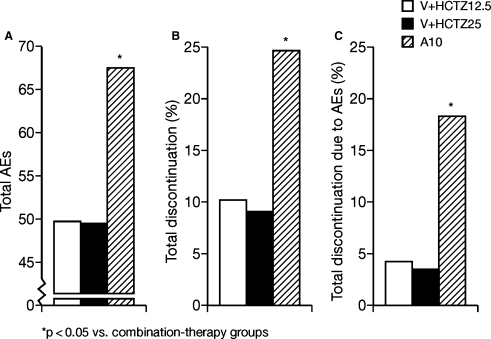
Rates of (A) total AEs, (B) total discontinuations and (C) total discontinuations as a result of AEs in the groups that received valsartan 160 mg plus hydrochlorothiazide 12.5 mg (V + HCTZ12.5), valsartan 160 mg plus HCTZ 25 mg (V + HCTZ2S) and amlodipine 10 mg (A10) (44)

Fixed-dose combinations of HCTZ and ARBs provide effective, simple, aggressive and well-tolerated BP control and are now rapidly gaining acceptance with physicians. The usefulness of ARB/HCTZ combinations in hypertension has now been demonstrated in clinical trials for most of the ARBs (42–47). Some of the most detailed recent data have been obtained with valsartan. In a recent double-blind, multicenter study of 24 weeks duration in 1088 patients, fixed-dose combinations of valsartan 160 mg with HCTZ 15.5 or 25 mg were found to reduce BP to a similar degree to amlodipine 10 mg (44). However, adverse events were significantly less frequent with the ARB/HCTZ combination and discontinuation rates as a result of adverse events were 4.2%, 3.5% and 18.2% in the valsartan/HCTZ 12.5 mg, valsartan/HCTZ 25 mg and amlodipine groups respectively (Figure 1). These BP-lowering and tolerability benefits of valsartan have also been reported to be sustained into the long term: in a recent open-label extension to a 1346-patient study with valsartan, sustained BP reductions were observed for 1 year, with mean BP reduced by 24.7/16.6 mmHg in patient receiving valsartan 320 mg/HCTZ 25 mg (48). Moreover, the incidence of hypokalaemia was significantly reduced compared with HCTZ monotherapy.

In the following section, the data for another representative ARB, irbesartan, are reviewed to explore in greater detail the clinical potential of the ARB/HCTZ strategy.

## Irbesartan/HCTZ trials

The safety and BP-lowering effect of the combination of irbesartan plus HCTZ, administered orally once a day, in patients with hypertension have been investigated in several clinical trials (Table 1). The irbesartan/HCTZ combination has been studied using various doses of each component or as a fixed dose given in a single tablet. In one study, patients with hypertension who were non-responsive to 4-week treatment with HCTZ 25 mg continued with HCTZ and were randomised to receive either irbesartan or matching placebo for 12 weeks (49). Irbesartan was given initially at a dose of 75 mg, doubled to 150 mg after 6 weeks for patients with diastolic BP ≥ 90 mmHg. Significant prompt reductions in BP appeared within 2 weeks of adding irbesartan. Furthermore at week 12, significantly more patients were normalised with irbesartan/HCTZ (67%) compared with placebo/HCTZ (29%).

**Table 1:** Studies of irbesartan/HCTZ in patients with essential hypertension

Study	Design/patients/dosage/duration	*n* (evaluable for efficacy)	Reduction in SDP/DBP at end-point	Response rates at end-point
Randomised blindedstudies				
Rosenstock 1998 (49)	Single blind, placebo-controlled Patients unresponsive to HCTZ Irbesartan 150–300 mg HCTZ 25 mg 12 weeks	238 (irbesartan 118,placebo 120)	11.1/7.2 mmHg (vs. placebo;p < 0.01)	67% (vs. 29% placebo;p < 0.01)
Kochar 1999 (45)	Double-blind, placebo-controlled,matrix design Mild-to-moderate hypertension Irbesartan 0, 37.5, 100 or 300 mg HCTZ 0, 6.25,12.5 or 25 mg 8 weeks	630 (300/25 mg,*n* = 41)	23.1/14.4 mmHg (300/25 mg)	44–80% withincreasing dose
Bobrie 2005(COSIMA study) (52)	Double-blind, comparative Untreated or uncontrolled hypertensives Irbesartan/HCTZ 150/12.5 mg Valsartan/HCTZ 80/12.5 mg 8 weeks	449 (irbesartan 222,valsartan 227)	Irbesartan 14/10.3 mmHg Valsartan 11.9/8.4 mmHg(Evening measurements,home BP monitoring)	50.2% (irbesartan) 33.2% (valsartan)(p < 0.01; normalised;home BP monitoring)
Neutel 2006 (61)	Double-blind, comparative Severe hypertension Forced titration Irbesartan/HCTZ 300/25 mg Irbesartan monotherapy 300 mg 7 weeks	737 (irbesartan/HCTZ 468,irbesartan 269)	9.7/4.7 mmHg (combinationtherapy vs. monotherapy;p < 0.0001)	47.2% (combinationtherapy) 33.2% (monotherapy)p = 0.0005; seatedDBP < 90 mmHg)
Selected non-blinded studies				
Raskin 1999 (50)	Open-label extension of two randomised,double-blind trials Mild-to-moderate hypertension Irbesartan/HCTZ 75/12.5–300/25 mg 1 year	1098	20.6/15.6 mmHg	83% normalised 90% responded 70% achieved specifiedBP goals
Neutel 2005(INCLUSIVE study) (61)	Open-label study Hypertensives with uncontrolled systolicblood pressure Irbesartan/HCTZ 150/12.5 mg–300/25 mg 16 weeks (after 4–5 weeks placebo & 2 weeks HCTZ run-in)	844	21.5/10.4 mmHg	69% achieved specifiedBP goals
Coca 2003 (59)	Open-label study Hypertension uncontrolled bymonotherapy/low-dosecombination therapy Irbesartan/HCTZ 300/25 mg 12 weeks	57	25.2/14.7 mmHg (ambulatoryBP measurements,peak levels)	94.7% SBP responders 87.7% DBP responders

The long-term safety and antihypertensive effects of irbesartan/HCTZ have also been demonstrated (50). Hypertensive patients completing two randomised, double-blind trials of irbesartan alone, HCTZ alone, irbesartan/HCTZ or placebo, received irbesartan 75 mg/HCTZ 12.5 mg once daily titrated to 150 mg/HCTZ 12.5 mg then if necessary 300 mg/HCTZ 25 mg until BP goals were achieved. If necessary, adjunctive therapies were added. From months 2 to 12, normalisation rates ranged from 75% to 85% and total responder rates ranged from 81% to 91%, while target BP was achieved in 65–75% of patients. At all time-points, most patients (> 87%) were receiving irbesartan/HCTZ alone. There were no reports of serious adverse events related to study medication.

Another study used a 4 × 4 factorial design or ‘matrix’ design to evaluate irbesartan and HCTZ across their respective dose ranges in patients with mild-to-moderate hypertension (45). A total of 683 patients were randomised to receive once-daily dosing with one of the 16 different double-blind, fixed combinations of irbesartan (0, 37.5, 100 and 300 mg) and HCTZ (0, 6.25, 12.5 and 25 mg). Mean changes from baseline in trough diastolic BP ranged from −3.5 mmHg for placebo, −7.1 to −10.2 mmHg for irbesartan monotherapy groups, −5.1 to −8.3 mmHg for HCTZ monotherapy groups and −8.1 to −15.0 mmHg for combination therapy groups. Importantly, irbesartan plus HCTZ produced additive reductions in BP, with at least one combination producing greater BP reduction than would be expected from the combination of both drugs (p < 0.001). Furthermore, as with other ARB studies, irbesartan tended to ameliorate the dose-related biochemical abnormalities associated with HCTZ alone. No dose-related adverse events were observed, and the incidence of adverse events and rates of discontinuation were comparable between treatment groups. The authors concluded that the combination of HCTZ in doses up to 25 mg and irbesartan in doses up to 300 mg is safe and produces dose-dependent reductions in BP.

### Difficult-to-treat patients and severe hypertension

**Figure 2:**
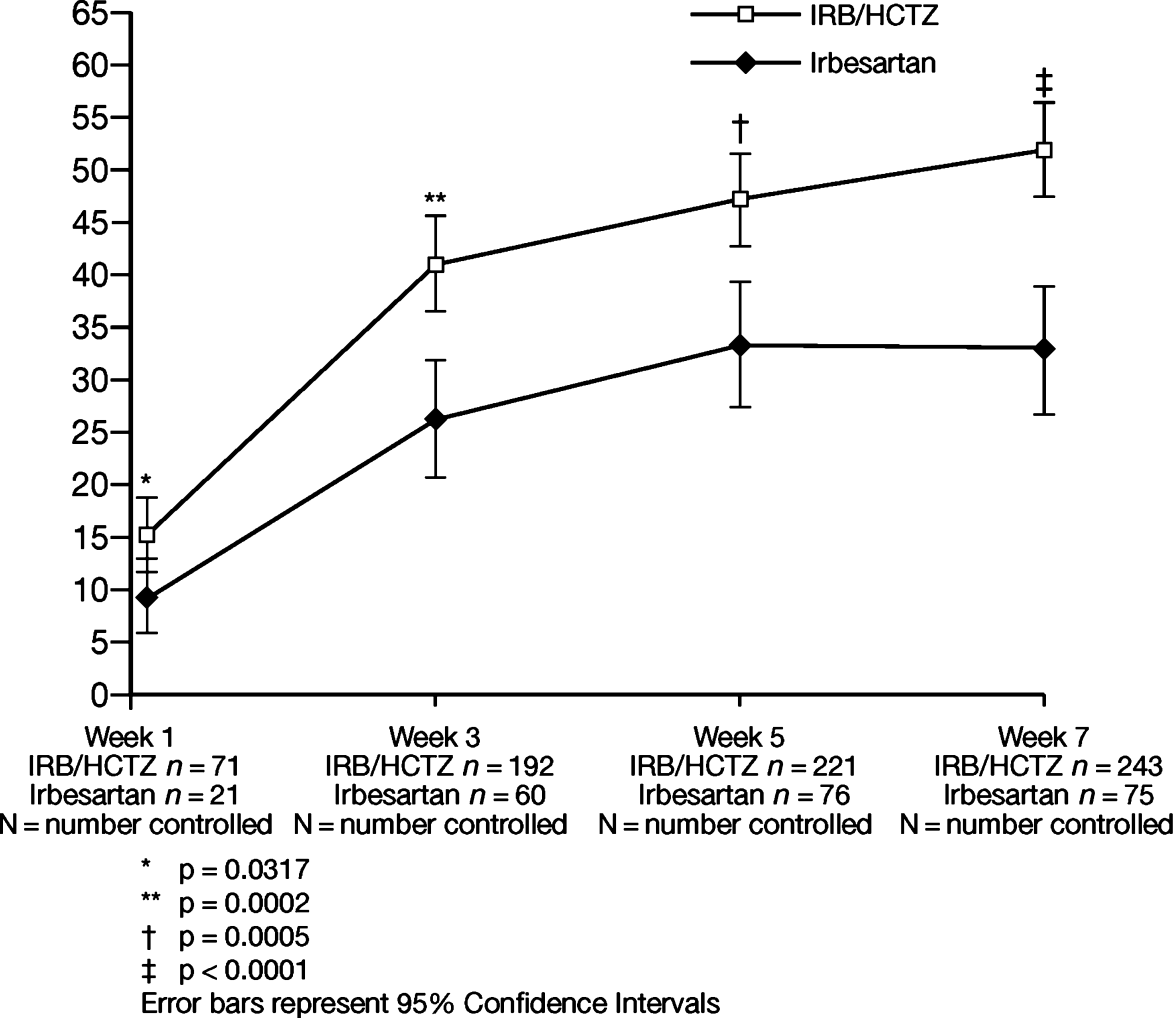
Comparison of the antihypertensive effects of irbesartan/HCTZ (150/12.5 mg) and valsartan/HCTZ (80/12.5 mg) in hypertensive patients: the COSIMA study (52). Reprinted with permission from the American Journal of Hypertension, Ltd

The combination of an ARB and HCTZ has been reported to be effective in difficult-to-treat and severely hypertensive patients for several ARBs, including losartan, candesartan, telmisartan and eprosartan (55–58). A high fixed-dose combination of irbesartan 300 mg/HCTZ 25 mg given once daily was also effective and well tolerated in patients with previously uncontrolled hypertension (59). As well as significantly reducing both clinic and ambulatory BP, 12 weeks of treatment preserved the circadian profile as shown by trough-to-peak ratios and smoothness index values for both systolic BP and diastolic BP (Figure 2). No metabolic changes were observed at these doses, and no patient discontinued the study because of treatment-related side effects.

The IrbesartaN/HCTZ bLood pressUre reductionS in dIVErse populations (INCLUSIVE) trial was a multicenter, prospective, open-label, single arm study to evaluate the BP-lowering efficacy and safety of a fixed-dose combination of irbesartan and HCTZ, at low dose (150 mg/12.5 mg) and at high dose (300 mg/25 mg) sequentially for 8 weeks each. Each combination of irbesartan and HCTZ was administered as a single tablet. Treatment was given to over 800 difficult-to-treat hypertensive patients including the elderly people, African-Americans, Hispanics, patients with type 2 diabetes and patients with the metabolic syndrome (60). Overall, 77% of patients achieved systolic BP control, and 83% achieved diastolic BP control after 8–16 weeks of treatment. The mean change in diastolic BP from baseline to the end of treatment was −10.4 ± 8.7 mmHg (p < 0.001) and the mean change in systolic BP was −21.5 ± 14.3 mmHg (p < 0.001). Further subanalyses showed that 96% of elderly patients achieved diastolic BP control and 73% achieved systolic BP. Over 70% of the elderly people, African-Americans, Hispanic/Latinos, those with metabolic syndrome, women and men achieved both systolic and diastolic BP control (60). Combination therapy was well tolerated and the results consistent across diverse patient populations.

**Figure 3:**
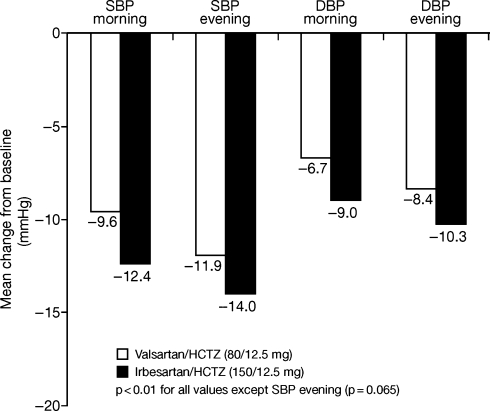
Percentage of patients achieving SeDBP < 90 mmHg during 7 weeks’ double-blind treatment of irbesartan/HCTZ combination therapy vs. irbesartan monotherapy (61)

Irbesartan has also been investigated in severely hypertensive patients. In a randomised, double-blind study of 737 patients with severe hypertension, 468 patients were given fixed-dose irbesartan/HCTZ 150/12.5 mg for 1 week then were force-titrated to a 300/25 mg dose for a further 6 weeks (61). The remaining 269 patients received irbesartan 150 mg monotherapy force-titrated to 300 mg monotherapy. At week 5, significantly more patients on combination therapy achieved the primary end-point of a seated DBP < 90 mmHg (47.2% vs. 33.2% respectively; p = 0.0005). More patients also reached the JNC-7 goal of < 140/90 mmHg at week 5 (34.6% vs. 19.2% respectively; p < 0.0001). Importantly, the rate at which BP control was achieved was also significantly more rapid in the irbesartan group (Figure 3). These effects were achieved without additional side effects.

## Comparative studies

Angiotensin receptor blockers used in monotherapy have been widely reported to vary in their BP-lowering efficacy. For instance, irbesartan at a starting dose of 150 mg has been reported to reduce BP more effectively than valsartan at its original starting dose of 80 mg (51). However, reported differences between ARBs generally reflect disparities between the doses selected for study. While the potency of individual molecules within the ARB class may indeed vary, with appropriate dosing their antihypertensive efficacy is broadly similar. In the aftermath of the VALUE trial, higher doses of valsartan are increasingly being used, and a starting dose of 160 mg would now be considered more appropriate than the 80 mg used in most comparative trials published to date.

Similar differences in BP-lowering efficacy have been reported in the fixed-dose combination setting, although the same difficulties with dose selection exist. The recent COmparative Study of efficacy of Irbesartan/HCTZ with valsartan/HCTZ using home blood pressure Monitoring in the treAtment of mild-to-moderate hypertension (COSIMA) study demonstrated greater antihypertensive efficacy of the fixed-dose combination of irbesartan 150 mg/HCTZ 12.5 mg vs. a fixed-dose combination of valsartan 80 mg/HCTZ 12.5 mg in an 8-week study of 414 hypertensive patients uncontrolled on HCTZ (52), although with a higher dose of valsartan greater parity might have been expected.

Differences in antihypertensive efficacy have also been reported between various other ARB/HCTZ combinations. For instance, in a recent 8-week study in 1066 patients, telmisartan/HCTZ 80/25 mg was reported to reduce both systolic and diastolic BP to a greater degree than valsartan/HCTZ 160/25 mg (53). Once again, however, it should be noted that telmisartan was used at its current maximum dose, whereas valsartan's maximum dose is now 320 mg. In a smaller study in 130 patients with hypertension uncontrolled by monotherapy, the addition of HCTZ 12.5 mg to valsartan 160 mg was recently reported to lead to greater additional reductions in BP than when added to olmesartan 20 mg (54); however, since both ARBs were being used at different starting doses, these results are difficult to evaluate.

In conclusion, it remains difficult to rank ARB/HCTZ combinations in terms of basic BP-reducing efficacy, as relatively few head-to-head trials have been carried out and the majority have not compared agents at like-for-like doses and with respect to universally agreed end-points. The goal of treatment should therefore be to treat patients to target irrespective of which particular ARB/HCTZ combination is used, with prompt dose escalation and the addition of a third agent whenever necessary to achieve this goal.

## Irbesartan reduces albuminuria independent of blood pressure lowering

This study highlights an intriguing aspect of therapy with angiotensin receptor blockade. That is, the dose–response curves for BP-lowering and target-organ protection are not identical. In this double-masked, randomised, crossover study in 52 hypertensive persons with type 2 diabetes and microalbuminuria, all treated with bendroflumethiazide 5 mg/day, irbesartan was also administered in doses of 300, 600, and 900 mg/day (62). Urinary albumin excretion was lowered an additional 15% more with 900 mg/day of irbesartan compared with lower doses; ambulatory SBP was lowered by 8, 9, and 9 mmHg, respectively, with higher irbesartan doses. These data show incremental target-organ protection with doses of irbesartan much higher than the FDA-approved dose for hypertension (300 mg/day) that appears distinct from the magnitude of BP lowering.

## Conclusions

There is a need for effective, safe and simple therapies to treat hypertension to recommended BP targets rapidly and rigorously, but with good tolerability and sustained patient adherence. The use of combination therapy as first-line treatment will help more patients promptly achieve BP goals, and fixed-dose combinations provide a means for simple but flexible dosing. Combination therapy using ARBs with HCTZ provides greater potency and fewer side effects than higher-dose monotherapy with either agent, and potentially offers benefits beyond those of BP lowering alone, particularly in high-risk hypertensives. The results from the irbesartan/HCTZ studies illustrate the potential of ARB/HCTZ therapy as a starting treatment in patients with moderate and severe hypertension; in patients with stage 2 disease such treatment enables management goals specified by current guidelines to be more rapidly and effectively achieved.
